# Associations Between Social Vulnerability and Providing Evidence-Based Diabetes Prevention and Management Activities in South Carolina, 2019

**DOI:** 10.5888/pcd20.220199

**Published:** 2023-02-23

**Authors:** Jennifer Mandelbaum, Courtney Brightharp, Kristian Myers, Shauna Hicks

**Affiliations:** 1 Division of Diabetes and Heart Disease Management, South Carolina Department of Health and Environmental Control, Columbia, South Carolina

## Abstract

We assessed associations between social vulnerability (ie, external stressors negatively affecting communities) and the provision of evidence-based diabetes prevention and management activities (eg, National Diabetes Prevention Program) in South Carolina counties with high burdens of diabetes and heart disease. These associations were examined by using relative risk estimation by Poisson regression with robust error variance. Results suggest that social vulnerability may have differential effects on the provision of evidence-based diabetes prevention and management activities in South Carolina. Findings support calls to identify upstream social factors contributing to adverse health outcomes and provide several potential points for intervention.

**Summary**

**What is already known on this topic?**

Evidence-based strategies to improve diabetes outcomes exist (eg, the National Diabetes Prevention Program), yet little is known about how providing these chronic disease prevention and management activities may differ by social vulnerability (ie, external stressors negatively affecting communities).

**What is added by this report?**

We assessed associations between social vulnerability and providing evidence-based diabetes prevention and management activities in South Carolina counties with high burdens of diabetes and heart disease. Results suggest that social vulnerability may affect the provision of evidence-based diabetes prevention and management activities at Rural Health Clinics and Federally Qualified Health Centers.

**What are the implications for public health practice?**

Findings support calls to identify upstream social factors contributing to adverse health outcomes and provide several potential points for intervention (eg, supporting collaboration between Rural Health Clinics, Federally Qualified Health Centers, and community-based pharmacists to facilitate the National Diabetes Prevention Program and medication therapy management).

## Objective

In South Carolina, 13.0% of adults have diabetes and 34.9% of adults have prediabetes (1,2). Several evidence-based strategies to improve diabetes outcomes exist, including the National Diabetes Prevention Program (NDPP) (3), diabetes self-management education and support (DSMES) (4), and medication therapy management (5). Two important sources of diabetes care in South Carolina are Rural Health Clinics (RHCs) and Federally Qualified Health Centers (FQHCs). These health care practices are safety net providers offering primary care (eg, chronic care and preventive health services) in rural and medically underserved areas (6). Although RHCs and FQHCs serve similar populations, they differ in terms of size, services offered, and funding. FQHCs are typically larger than RHCs, and they may have multiple sites and offer specialty care, such as dental services. FQHCs also receive federal funding and must, therefore, comply with requirements from the Health Resources and Services Administration (7). Little is known about how providing these chronic disease prevention and management activities may differ by social vulnerability (ie, external stressors negatively affecting communities) (8,9). This study assessed associations between social vulnerability and providing evidence-based diabetes prevention and management programs at RHCs and FQHCs in South Carolina counties with high burdens (death, hospitalizations, and prevalence) of diabetes and heart disease.

## Methods

Data on diabetes prevention and management activities came from a cross-sectional survey of RHCs and FQHCs (N = 71) in the top 50% of South Carolina counties with the highest burden of diabetes and heart disease. The sample was determined by county-level standardized *z* scores of diabetes and heart disease burden, weighted as mortality (50%), hospitalizations (30%), and prevalence (20%) for both conditions. RHCs and FQHCs providing only pediatric, behavioral health, gynecologic/obstetric, dental, or pharmacy services were excluded. Health care practices completed the online survey during February through August 2019. FQHCs include several individual health care practices; representatives from 7 FQHCs answered survey questions on behalf of 50 total practices. The decision to have these representatives respond for the individual practices was made in consultation with local partners and 2 statewide associations, the South Carolina Office of Rural Health (SCORH) and the South Carolina Primary Health Care Association (SCPHCA) to reduce the administrative burden on FQHCs. SCORH and SCPHCA assisted with survey distribution and data validation.

The survey response rate was 78%. Data represented 21 RHCs and 50 FQHCs and included 22 of 46 counties in the state (Figure). These counties were primarily in the Interstate 95 corridor, a 200-mile stretch of highway along the coastal plain of South Carolina that is home to nearly 25% of South Carolinians (10). Survey responses reflect services provided by practices within the previous year. Survey methodology and sample statistics are published elsewhere (6). Sites were asked if they implemented evidence-based activities including NDPP and medication therapy management for patients with prediabetes, DSMES or medication therapy management for patients with diabetes, and the Diabetes Prevention Toolkit for Physicians and Health Care Teams to evaluate, test, or treat patients with diabetes (11). Activities at each site were coded as 0 (activity not offered) or 1 (activity offered).

**Figure:**
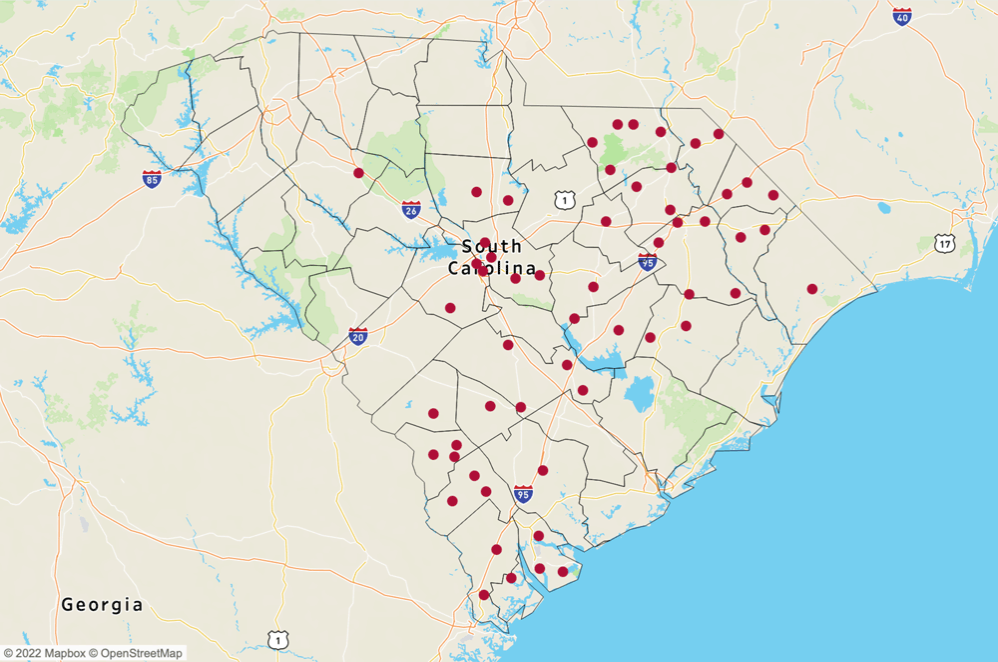
Location of 21 Rural Health Clinics (RHCs) and 50 Federally Qualified Health Centers (FQHCs) in 22 counties in South Carolina, 2019.

County-level social vulnerability measures were extracted from the Centers for Disease Control and Prevention’s 2018 Minority Health Social Vulnerability Index (SVI) (9,12). Specific measures in the SVI came from sources including the American Community Survey and the Homeland Infrastructure Foundation-Level Data Open Data (9). The SVI is based on 34 census variables covering 6 themes: socioeconomic status, household composition and disability, minority status and language, housing type and transportation, health care infrastructure, and medical vulnerability. Each theme comprised 4 to 11 indicators. For example, social vulnerability related to socioeconomic status encompassed the percentage of persons who live below poverty guidelines, the percentage of persons who were unemployed, per capita annual income, and the percentage of persons aged 25 years or older with no high school diploma. SVI was measured as a percentile ranking, which ranged from 0 (least vulnerable) to 1 (most vulnerable) (12).

Data were analyzed by using Stata version 15 (StataCorp LLC). To reduce confounding and the potential for reverse causality, the analysis did not include social vulnerability related to medical vulnerability (because the sample of counties was chosen on the basis of diabetes and heart disease burden). Descriptive statistics (means, frequencies) were calculated for 7 evidence-based diabetes prevention and management activities (implemented NDPP; implemented DSMES; offered medication therapy management for prediabetes; offered medication therapy management for diabetes; and used the diabetes prevention toolkit to evaluate, test, or treat patients for diabetes) and SVI measures and their indicators. Associations between each SVI measure and each of 7 evidence-based activities were examined by using relative risk estimation by Poisson regression with robust error variance. The multilevel analysis accounted for clustering at the county and center levels.

## Results

About half of health care practices reported implementing the NDPP (51%), and about one-third of practices reported implementing DSMES (34%; Table 1). Nearly half of practices reported offering medication therapy management for diabetes (48%), while about one-quarter of sites reported offering medication therapy management for prediabetes (25%). About one-third of sites reported using the diabetes prevention toolkit to evaluate (34%), test (35%), or treat (35%) patients with diabetes. Social vulnerability percentiles for each theme ranged from 0.51 (social vulnerability related to health care infrastructure) to 0.79 (social vulnerability related to housing type and transportation).

**Table 1:** Activities Offered by Health Care Practices and Social Vulnerability in South Carolina Counties With the Highest Burden of Diabetes and Heart Disease (N = 71), 2019

Characteristic	Value
**Baseline assessment measures, n (%)**	
Implemented NDPP	36 (51)
Implemented DSMES	24 (34)
Offered medication therapy management for prediabetes	18 (25)
Offered medication therapy management for diabetes	34 (48)
Used diabetes prevention toolkit to evaluate patients for diabetes	24 (34)
Used diabetes prevention toolkit to test patients for diabetes	25 (35)
Used diabetes prevention toolkit to treat patients for diabetes	25 (35)
**Social vulnerability measures^a^ related to:**	
Socioeconomic status	0.76 (0.26)
Persons below poverty, % (SD)	20.86 (5.40)
Persons unemployed, % (SD)	8.68 (2.43)
Per capita annual income, mean (SD), $	23,273.00 (5,990.85)
Persons aged ≥25 y with no high school diploma, % (SD)	16.03 (5.50)
Household composition and disability	0.64 (0.28)
Persons aged 65 y or older, % (SD)	17.81 (3.52)
Persons aged 17 y or younger, % (SD)	21.85 (1.80)
Civilian noninstitutionalized population with a disability, % (SD)	17.08 (3.25)
Single parent household with children aged under 18 y, % (SD)	10.22 (1.98)
Minority status and language	0.64 (0.26)
African American or Black, % (SD)	45.42 (16.25)
American Indian and Alaska Native, % (SD)	0.48 (0.59)
Asian, % (SD)	0.96 (0.81)
Hispanic or Latino, % (SD)	4.05 (2.67)
Native Hawaiian/Pacific Islander, % (SD)	0.07 (0.09)
Some other race alone, % (SD)	1.20 (0.87)
Spanish speakers who speak English less than very well, % (SD)	1.51 (1.08)
Chinese speakers who speak English less than very well, % (SD)	0.07 (0.08)
Vietnamese speakers who speak English less than very well, % (SD)	0.05 (0.05)
Korean speakers who speak English less than very well, % (SD)	0.05 (0.09)
Russian speakers who speak English less than very well, % (SD)	0.02 (0.03)
Housing type and transportation	0.79 (0.18)
Multi-unit housing, % (SD)	4.89 (5.52)
Mobile homes, % (SD)	25.24 (11.32)
Crowded housing, % (SD)	2.16 (0.85)
Households with no vehicle available, % (SD)	8.92 (3.42)
Persons in group quarters, % (SD)	4.10 (3.19)
Health care infrastructure	0.51 (0.28)
Hospitals, mean no. (SD)	3.46 (2.02)
Urgent care clinics, mean no. (SD)	1.13 (1.46)
Pharmacies, mean no. (SD)	23.01 (5.58)
Primary care physicians per 100,000 population, mean no. (SD)	0.53 (0.26)
Persons without health insurance, % (SD)	11.36 (1.38)
Persons without internet access, % (SD)	28.12 (10.00)

Abbreviations: DSMES, diabetes self-management education and support; NDPP, National Diabetes Prevention Program.

a Defined as external stressors negatively affecting communities. Social vulnerability was measured a percentile ranking, which ranged from 0 (least vulnerable) to 1 (most vulnerable) (12).

No significant associations were found between social vulnerability related to housing type and transportation and providing evidence-based diabetes prevention and management activities (Table 2). Practices in areas with higher social vulnerability related to socioeconomic status and household composition and disability were less likely to report implementing the NDPP. For example, high social vulnerability was associated with a 60% lower likelihood that an RHC or FQHC implemented the NDPP (prevalence ratio [PR], 0.40; 95% CI, 0.23–0.69). Social vulnerability related to minority status and language was associated with decreased provision of medication therapy management for prediabetes (PR, 0.63; 95% CI, 0.46–0.85). Higher social vulnerability related to health care infrastructure (eg, hospitals per 100,000 population, persons without health insurance) was associated with increased provision of medication therapy management for prediabetes (PR, 1.48; 95% CI, 1.01–2.17) and diabetes (PR, 1.71; 95% CI, 1.09–2.71).

**Table 2:** Associations Between Social Vulnerability Measures and Diabetes Prevention and Management Activities at South Carolina Rural Health Clinics (RHCs) and Federally Qualified Health Centers (FQHCs) (N = 71), 2019

Social vulnerability measure^a^/diabetes activity	Prevalence ratio (95% CI)
**Socioeconomic status**	
Implemented NDPP	0.40 (0.23–0.69)^b^
Implemented DSMES	1.25 (0.30–5.15)
Offered medication therapy management for prediabetes	336.35 (6.83–16,564.09)
Offered medication therapy management for diabetes	1.36 (0.44–4.22)
Used diabetes prevention toolkit to evaluate patients for diabetes	1.01 (0.27–3.78)
Used diabetes prevention toolkit to test patients for diabetes	1.02 (0.26–3.95)
Used diabetes prevention toolkit to treat patients for diabetes	1.02 (0.26–3.95)
**Household composition and disability**	
Implemented NDPP	0.20 (0.04–0.93)^b^
Implemented DSMES	0.74 (0.08–6.69)
Offered medication therapy management for prediabetes	1.49 (0.66–3.38)
Offered medication therapy management for diabetes	1.27 (0.42–3.78)
Used diabetes prevention toolkit to evaluate patients for diabetes	1.00 (0.08–12.06)
Used diabetes prevention toolkit to test patients for diabetes	0.34 (0.04–3.24)
Used diabetes prevention toolkit to treat patients for diabetes	0.34 (0.04–3.24)
**Minority status and language**	
Implemented NDPP	2.55 (0.40–16.42)
Implemented DSMES	0.36 (0.09–1.41)
Offered medication therapy management for prediabetes	0.63 (0.46–0.85)^b^
Offered medication therapy management for diabetes	0.76 (0.53–1.08)
Used diabetes prevention toolkit to evaluate patients for diabetes	0.48 (0.08–2.75)
Used diabetes prevention toolkit to test patients for diabetes	0.56 (0.11–2.97)
Used diabetes prevention toolkit to treat patients for diabetes	0.56 (0.11–2.97)
**Housing type and transportation**	
Implemented NDPP	0.63 (0.25–1.60)
Implemented DSMES	1.37 (0.16–11.72)
Offered medication therapy management for prediabetes	1.60 (0.57–4.54)
Offered medication therapy management for diabetes	1.41 (0.45–4.48)
Used diabetes prevention toolkit to evaluate patients for diabetes	1.01 (0.05–21.76)
Used diabetes prevention toolkit to test patients for diabetes	0.56 (0.07–4.63)
Used diabetes prevention toolkit to treat patients for diabetes	0.56 (0.07–4.63)
**Health care infrastructure**	
Implemented NDPP	0.82 (0.56–1.20)
Implemented DSMES	1.26 (0.46–3.41)
Offered medication therapy management for prediabetes	1.48 (1.01–2.17)^b^
Offered medication therapy management for diabetes	1.71 (1.09–2.71)^b^
Used diabetes prevention toolkit to evaluate patients for diabetes	0.77 (0.24–2.49)
Used diabetes prevention toolkit to test patients for diabetes	0.78 (0.25–2.43)
Used diabetes prevention toolkit to treat patients for diabetes	0.78 (0.25–2.43)

Abbreviations: DSMES, diabetes self-management education and support; NDPP, National Diabetes Prevention Program.

a Defined as external stressors negatively affecting communities. Social vulnerability was measured a percentile ranking, which ranged from 0 (least vulnerable) to 1 (most vulnerable) (12).

b *P* < .05.

## Discussion

Results suggest that social vulnerability measures may have differential effects on the provision of evidence-based diabetes prevention and management activities at RHCs and FQHCs in South Carolina. Across themes, higher social vulnerability tended to be related to providing fewer diabetes activities, with one exception: higher social vulnerability related to health care infrastructure was associated with providing more medication therapy management. These findings may reflect, in part, the demographics and health care–associated vulnerabilities of the Interstate 95 corridor (10), where many of the RHCs and FQHCs in this sample were located. Findings highlight the need for future work to investigate providing medication therapy management; medication therapy management may have been offered as an alternative to more resource-intensive programs such as DSMES or NDPP.

One limitation of this study is RHC staff answered questions on behalf of their individual practice, whereas FQHC representatives completed the survey for all practices affiliated with their center. However, the multilevel analysis did account for clustering at the center level. Another limitation is that analyses grouped RHCs and FQHCs together. FQHCs may be more likely to have a pharmacist available and, therefore, have greater capacity to implement programs like NDPP and medication therapy management. Lastly, this study only included RHCs and FQHCs, while other medical facilities, county agencies, and community organizations may also offer evidence-based diabetes prevention and management activities.

Findings support calls to identify upstream social factors contributing to adverse health outcomes and provide several potential points for intervention (13). For example, interventions may support better collaboration between FQHCs, RHCs, and community-based pharmacists to facilitate NDPP and medication therapy management. Community-level efforts outside of RHCs and FQHCs might also address systemic factors related to low socioeconomic status, household composition, and language access. Currently, the state health department is piloting a new funding initiative in which community coalitions can apply for a grant to address social vulnerabilities that may prevent people with prediabetes from participating in the NDPP. This pilot program approaches health equity from the perspective of the systems providing health services, not from the patient perspective. Results from this study provide a baseline for future data collection, which could allow for tracking these associations over time.
